# Myasthenia gravis therapy with individualized homeopathy: A case report

**DOI:** 10.1002/ccr3.3190

**Published:** 2020-07-29

**Authors:** Vitalie Văcăraș, Cristina Nistor, Imelda Rahovan, Cristiana Văcăraş, George Vithoulkas

**Affiliations:** ^1^ Department of Neurosciences “Iuliu Hatieganu” University of Medicine and Pharmacy Cluj‐Napoca Romania; ^2^ Neurology Department Cluj County Emergency Clinical Hospital Cluj‐Napoca Romania; ^3^ Medical Student Iuliu Hatieganu” University of Medicine and Pharmacy Cluj‐Napoca Romania; ^4^ University of the Aegean Mytilene Greece

**Keywords:** homeopathy, myasthenia gravis, therapy

## Abstract

We present a 61‐year‐old man with severe myasthenia gravis, nonresponsive to conventional therapy. The patient was treated with individualized homeopathy, demonstrating significant improvement on his clinical status and no disease symptoms.

## Graphical abstract

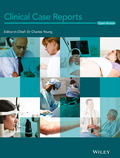

## 1 INTRODUCTION

Homeopathy can be considered a complementary treatment option for patients with a severe form of myasthenia gravis whose symptoms are not controlled with conventional medication. Individualized therapy is administered based on the levels of health theory.

Myasthenia gravis is an autoimmune pathology of neuromuscular transmission characterized by variable degrees of weakness in skeletal muscles.^1^ The global prevalence is 70‐320 cases per one million people, so it is considered a relatively rare disease.^2^ The age of onset often has a bimodal distribution: 20‐30 years old with female predominance and 70‐80 years old with male predominance.^3^

Up to 90% of patients diagnosed with myasthenia gravis have autoantibodies against the acetylcholine receptor (AChR) in their serum; these antibodies cause a major blockage of the neuromuscular junction.^4^ There are also patients who present with antibodies against muscle‐specific receptor tyrosine kinase (MuSK)^5^ and a small proportion of patients with neither of these antibodies (seronegative).^6^ The majority of patients are also diagnosed with thymic pathology, including hyperplasia and thymoma.^1^

The clinical aspects of myasthenia gravis are classified into an ocular form, characterized by a weakness limited to extraocular muscles and eyelids, and a generalized form, with variable involvement of bulbar, respiratory, and limb muscles.^7^ For the majority of patients, the disease onset is characterized by ocular symptoms; weakness of the eyelid muscles, leading to ptosis; and weakness of the extraocular muscles, leading to diplopia.^8^ Some patients may develop bulbar symptoms, such as dysphagia, dysarthria, and fatigable chewing. In rare cases, proximal limb weakness is the only manifestation.^7^ When the severe pathological process involves the respiratory muscles, weakness in these muscles can lead to respiratory failure and myasthenic crisis. This can be aggravated by different factors, such as infections, surgery, medications, or the natural course of the disease.^9^

The typical examinations that lead to the diagnosis of myasthenia gravis are laboratory methods (to find specific autoantibodies) and electrophysiologic studies (repetitive nerve stimulation and single‐fiber electromyography).^10^

The use of acetylcholinesterase inhibitors such as pyridostigmine may increase the amount of acetylcholine available at the neuromuscular junction. Glucocorticoids and immunosuppressive products represent a chronic treatment option. Immunomodulating agents such as plasma exchange and intravenous immune globulin are used in certain cases for rapid and short‐term effects.^11^ Muscle impairment may lead to respiratory failure, aspiration, pneumonia, falls, and mortality in approximately 3%‐4% of all cases.^12^

## 2 CASE HISTORY

### 2.1 Case presentation

We describe the case of a 61‐year‐old man who presented to the Neurology Department in April 2018 for sudden onset diplopia (predominantly on the left side) that would worsen in the evening and mild dysphonia. The patient had a medical history of various pathologies, such as arterial hypertension, deep venous thrombosis, gastric and duodenal ulcer, chronic bronchitis, uncontrolled allergy with severe pruritus, disk hernias, and hemorrhoids. For 14 years, he also suffered from recurrent prostatitis and severe urinary infections (at least one episode per year), with a high fever up to 40°C. The last episode (in 2016) was treated with antibiotics for 45 days, leading to a period of 2 years with no fever.

In April 2018, he presented with symptoms which were clinically suspected to be ocular myasthenia gravis and then rapidly confirmed by paraclinical tests. The electromyography showed myasthenic decrement; thymic computer tomography with contrast revealed no abnormalities; and acetylcholine receptor antibodies (AchR) were positive, with a value of 8.83 nmol/L (normal value <0.4 nmol/L). At that time, the patient received corticoid treatment with methylprednisolone (8 mg/day, with a gradual increase) and pyridostigmine (30 mg three times per day). His clinical status was improved.

Three weeks later, he presented with a myasthenic crisis with diplopia, bilateral ptosis, dysphagia, dysphonia, dysarthria, and acute respiratory failure. At onset, laboratory findings revealed hyponatremia 135 mmol/L (NV* 136‐146 mmol/L), hypokalemia 3.2 mmol/L (NV 3.5‐5.1 mmol/L), hypochloremia 98 mmol/L (NV 101‐109 mmol/L), leukocytosis 15.83 × 10^9^ (NV 4‐10 × 10^9^) with neutrophilia 12.3 × 10^9^ (NV 1.5‐6.6 × 10^9^). The blood analysis evolved with the worsening of the hydroelectrolitic balance (sodium level of 127 mmol/L), acute renal failure with creatinine 1.34 mg/dL (NV 0.67‐1.17 mg/dL) and urea 50 mg/dL (NV 17‐43 mg/dL), coagulopathy INR 1.33 (NV 0.8‐1.2) and the worsening of the inflammatory process, with a C reactive protein level of 19.22 mg/dL, a value up to 40 times higher than the normal one lower than 0.5 mg/dL, and a fibrinogen of 826.8 mg/dL (NV 200‐400 mg/dL). The investigations revealed severe pneumonia. After an intensive care treatment which included antibiotics (ceftriaxone 2 g/day), proper hydration (750 mL/day), intravenous immunoglobulin therapy (12 vials of 50 mL of human immunoglobulin solution for 5 days), pyridostigmine (60 mg four times per day), and azathioprine (50 mg/day), the patient showed clinical and biological improvements. He was discharged with a diagnosis of myasthenia gravis stage IIA; he still presented ptosis, diplopia, and mild swallowing deficits.

*NV = normal value.

### 2.2 Homeopathic treatment

After an acute decompensation in April 2018, the patient was treated with individualized homeopathic remedies complementary to conventional therapy. The treatment was chosen according to the classical homeopathy principles, as it is presented in Table 1. In this presented case, one dose of homeopathic treatment consists of seven granules of remedy administered sublingually.

**TABLE 1:** The series of homeopathic interventions

Date	Symptoms	Concomitant conventional therapy	Homeopathic prescription
8 Aug 2018	The patient presented initially with bilateral eye ptosis, mostly on the left side (d.2^a^), moderate dysphagia (d.2), and diplopia (d.2). He also declared anxiety about his health (d.2), major fear of death (d.4), sensation of imminent death (d.3), and panic attacks (d.3). Moreover, he expressed a fear of crowded places (d.3), fear of tunnels (d.2), aversion to sweets (d.2), preference for smoked meat (d.2), cold sensations (d.2), sleeping position on right side (d.2), intolerance for injustice (d.2), and sympathy (d.2).	Pyridostigmine 60 mg, 3 tb/day Azathioprine 50 mg, 1 tb/day	Aconitum napellus 30C one dose daily for 2 mo
7 Nov 2018	Anterior myasthenia gravis symptoms persisted, at the same intensity (eye ptosis, dysphagia, diplopia). The patient had an improved general status, more energetic. The fear of death and panic attacks disappeared. At similar intensities, some of the symptoms persisted: anxiety about health, desire for smoked meat, aversion to sweets, cold sensations, sleeping position on right side, sympathy, and intolerance for injustice (d.3). Recently, he started to have excessive salivation (d.2) and mouth inflammation (d.2).	Unchanged	Causticum 30C one dose daily for 3 mo, then 32C for 3 mo, followed by 34C for the next 3 mo
7 Nov 2019	The initial myasthenia gravis clinical aspects were absent, with no palpebral ptosis, dysphagia or diplopia. He presented with sudden onset fever (40°C), burning pain during urination (d.3), aggravation after urination (d.4) and frequent urination (d.3). He also presented with nocturnal perspiration (d.3), an increase in anxiety after perspiration (d.2), a major sensation of cold (d.3), and felt worse during the night (d.3). The patient did not receive antibiotics during the infection stage.	Unchanged	Mercurius corrosivus 30C twice a day for 1 wk, then one dose daily for 1 mo
19 Feb 2020	The patient had a high level of well‐being, with no clinical signs of myasthenia gravis. After Causticum treatment, the hemorrhoids from his medical history reappeared. Additionally, a face allergy that was treated some years ago very aggressively with medication reappeared, with intense pruritus. He had a persistent fear of suffering (d.3), anxiety about health problems (d.3). Additionally, he became extremely neat (d.3), with a precise order of thoughts, and he became critical of other people (d.2). The sensation of cold remained persistent. He had a desire for fatty food and he drank water with small sips (d.3). However, he started eating sweets more often.	Pyridostigmine 60 mg, 3 tb/day Begin to decrease the dose of Azathioprine 50 mg to ¼ tb/day	Arsenicum album 30C 1 dose daily for 3 wk
15 Mar 2020	The myasthenia gravis symptoms were still absent; there was no clinical sign of the disease. The face allergy and hemorrhoids also disappeared. The patient presented no fear about health problems as he did on the last visit.	Pyridostigmine 60 mg, 2 tb/day, with gradual lowering of the dose	No remedy prescribed

^a^ d, degree, a measurement for the intensity of the symptom.

### 2.3 Outcome

In combination with conventional medication, the patient was treated with a series of individualized homeopathic remedies, chosen according to his symptoms presented at each follow‐up visit.

After the last hospitalization, while being treated with pyridostigmine and azathioprine, the patient maintained a steady clinical status but presented with persistent clinical signs of myasthenia gravis, including palpebral ptosis, dysphagia, diplopia, and AchR antibodies at a value of 8.83 nmol/L (normal range <0.4 nmol/L). At that time, after the first homeopathic consultation, he was prescribed Aconitum napellus.

In 3 months, the patient gained more energy and strength, with no more panic attacks or fear of death, although dysphagia, diplopia, and ptosis still persisted at the same intensity. He started treatment with Causticum.

After 1 year, at the next visit, he developed an acute urinary infection and prostatitis, with pronounced symptoms and fever. His prostate‐specific antigen (PSA) value was 49.8 ng/mL, which is more than 12 times higher than the normal value of 4.1 ng/mL, associated with leukocytosis with neutrophilia and lymphocytosis. The initial myasthenia gravis symptoms were completely absent at this point. The patient did not agree to receive any antibiotic treatment at that time. He started treatment with Mercurius corrosivus, and in 1 week, the inflammatory syndrome was absent. He had a progressive lowering of the PSA value (a change up to 6 ng/mL over 1 month). The acute episode of infection was absent.

The fourth follow‐up visit after 3 months revealed no signs of inflammation, and the myasthenia gravis signs were still absent; therefore, we started lowering the dosage of azathioprine to 12.5 mg/day. A previously treated allergy reappeared along with hemorrhoids. He was prescribed Arsenicum album.

In 1 month, the allergy and hemorrhoids also disappeared, the patient declared a state of well‐being, and there were no more remedies prescribed. He had no clinical signs of myasthenia gravis on neurological examination and no symptoms; only the remaining AchR antibodies fluctuated over time, from 8.83 nmol/L at the beginning to 5.11 nmol/L after 1 year. He stopped the azathioprine and started progressively lowering the dosage of pyridostigmine, until he completely stopped using conventional medication. According to his good clinical status, he does not take at present any homeopathic treatment either.

## 3 DISCUSSION

Homeopathy is a therapeutic approach of medicine that uses substances selected from nature, such as minerals, chemicals, and plants, which are diluted and potentiated.^13^ Its aim is to restore the internal order by stimulating the patient's defense mechanisms.^14^

Homeopathic approaches are based on the levels of health theory, which implies that each person has a specific energy needed for all vital functions and health maintenance, and this energy is influenced by genetics, environment, thoughts, and treatments. People with a low level of health are more difficult to treat or are less likely to present with a systematic improvement until they are treated with the correct successive remedies for a long period of time; those with a higher level of health can be completely cured in a faster and easier manner.^15^

The energy complex of the organism is the capacity of the body to react to overall stimuli, and the symptoms produced by the defense mechanism may appear to protect the inner vital organs. Hahnemann's law of cure states that the symptoms of the remedy and the symptoms produced by the defense mechanism of a patient in a disease state are similar.^14^

A homeopathic treatment uses a source substance and passes through a dilution process (potentization) in which the substance is still biologically active by retaining the proprieties of the initial material .^16^ High potencies are obtained by dilution factors greater than the Avogadro's number (<10^23^). Although it was thought that these dilutions could not contain any active ingredients, recent studies have proven otherwise by detecting the presence of substances in the form of nanoparticles of the initial material by transmission electron microscopy and electron diffraction.^17^ In homeopathy, a more diluted solution is considered to have a higher potency.^15^ The potency choice is accordingly also related to the health level: a lower health level or a state of imminent danger implies a low potency, such as 30C. ^14^

Our patient was included in a low health level of 7, group C. This level contains all degenerative chronic diseases, where the disturbed immune system is not capable of properly reacting to foreign agents with high fever. If we suppress repeated acute conditions, the underlying hereditary pathology is activated and leads to a serious chronic disease.^14^ Our patient had a full medical history, including urinary infections with high fever that was suppressed by aggressive medication for a period of time. In such periods, the organism is only in an apparent healing state, but it is a prodrome for more serious chronic diseases—in his case, myasthenia gravis. Patients with a low energy level do not have a classical clinical pattern and can be treated only with a series of well‐chosen remedies; therefore, we started with the most common remedy, that is, Aconitum napellus, because of his major fear of death.

The healing process of a homeopathic treatment can be observed in the inversion of the chronological pattern in which the symptoms appeared. The curative process has a direction from center to periphery, meaning that the symptoms should move from vital to less important organs and from a mental state to a physical one.^18^ After the first remedy, our patient started feeling better. The second treatment, Causticum, was followed by the reappearance of a former severe urinary infection and prostatitis, with high fever, which indicates the reactivation of normal defense mechanisms. An acute event during homeopathic treatment should be carefully evaluated, and it is a difficult moment for the therapist because he has to make a choice regarding the therapy: whether to continue with the same remedy, to wait and closely monitor the case or to find another proper treatment. An acute fever attack is the sign that the immune system allows the organism to properly react to infectious agents.^15^ In the case of our patient, some symptoms from the past reappeared in inverse order, firstly, urinary infection and prostatitis. In general, a patient who previously suppressed his acute febrile disease with allopath medication will develop a reactivation of his defense mechanism through fever, if he is under the correct homeopathic treatment. Also, in the case of our patient, the acute attack reappeared only after 1 year of multiple homeopathic remedies, which is highly suggestive for a low level of health. If the defense mechanisms are compromised, the acute phase will take place later on.^15^ The initial myasthenia gravis symptoms started to disappear. In our case, the clinical pattern was suggestive for Mercurius corrosivus, and after the treatment, the inflammation disappeared, with no other allopathic medicine. Some other former minor pathologies which had been suppressed temporarily reappeared, meaning that the organism was on its path to a better vital force.

At the last medical visit, our patient had no sign of myasthenia gravis, except for the presence of AchR antibodies. He presented with an excellent clinical status and psychological health.

Myasthenia gravis is a common disorder of neuromuscular transmission. Although most of the patients respond to conventional medicine, some cases are refractory to medication, and thus, treatment options remain limited.^19^ Our patient was treated with a homeopathic treatment in parallel with an allopathic one and had a good outcome; he presented significant improvements, leading to the progressive decreasing of the conventional treatment dosages and to a good health.

## 4 CONCLUSION

We present the case of a 61‐year‐old man diagnosed with severe myasthenia gravis initially treated only with conventional therapy. After an individualized series of homeopathic remedies, he presented a good clinical outcome, with no clinical signs of the disease. This is one of the first cases of myasthenia gravis treated with homeopathy presented in the literature. Homeopathy is a useful complementary approach to be considered among patients with myasthenia gravis.

## CONFLICT OF INTEREST

The authors declare no conflicts of interest.

## AUTHOR CONTRIBUTIONS

VV: served as the principal author, collected and analyzed the data, and wrote the article. NC, RI, and VC: collected the data and contributed to the writing process. VG: provided guidance and supervised the manuscript.

## ETHICAL APPROVAL

The ethical principles in this case were applied.
